# The international data governance landscape

**DOI:** 10.1093/jlb/lsac005

**Published:** 2022-04-04

**Authors:** Alexander Bernier, Fruzsina Molnár-Gábor, Bartha Maria Knoppers

**Affiliations:** 1 Department of Human Genetics, McGill University Faculty of Medicine, Centre of Genomics and Policy, Montreal, Canada; 2 Heidelberg University, Faculty of Law, Heidelberg, Germany; 3 Heidelberg Academy of Sciences and Humanities, Heidelberg, Germany; 4 Department of Human Genetics, McGill University, Human Genetics, Montreal, Canada

**Keywords:** International data sharing, data protection law, biomedical ethics, health research policy, big data, biomedical data commons

## Abstract

As the adoption of digital health accelerates health research increasingly relies on large quantities of biomedical data. Research institutions scattered across a large number of jurisdictions collaborate in producing and analyzing biomedical big data. National data protection legislation, for its part, grows increasingly complex and localized. To respond to heterogeneous legal requirements arising in numerous jurisdictions, decentralized health consortia must develop scalable organizational and 6 technological arrangements that enable data flows across jurisdictional boundaries. In this article, proposals are made to enable health sector organisations to align established biomedical ethics process and data analysis practices to shifting data protection norms through both public law co-regulation, private law tools, and design-oriented approaches.

## I. INTRODUCTION

The biomedical research consortium is an organizational structure used to facilitate and promote research amongst groups of researchers that are dispersed throughout academic centers and clinical research institutions without a common territorial locus. This structure is adopted because researchers with common interests and compatible expertise are often established in unrelated and geographically distant research institutions. The structure is also useful because it allows researchers to collaborate in the generation, curation, analysis, and preservation of biomedical data for future use, allowing for the data created to serve as a shared source of knowledge for biomedical research.^1^

Numerous international research consortia have developed a sophisticated architecture of organizational bodies and technical infrastructure to provide access to their stewarded data both to internal collaborators and to external collaborators. Such infrastructure includes technological elements, such as online portals for data access and cloud-compute resources to help store and analyze data.^2^ Consortia also implement ‘policy’ infrastructure—including governance bodies, consortium policies regarding data inclusion criteria, and common procedures to access shared data.^3^ Model contracts or model informed consent materials are also adopted to streamline compatible data collection and data sharing activities. Consortia that have adopted these approaches to data sharing include the UK Biobank, the International Cancer Genome Consortium (ICGC), the Human Cell Atlas (HCA), and the H3 Africa Consortium.

These infrastructures for data exchange engage in constant dialogue with local, national, and supranational legal and ethical frameworks that determine how data can and cannot be utilized. In part, the consortium responds to the law and to research ethics in facilitating compliance with such regimes through the pooling of compliance expertise and compliance tools. At the same time, changes in legal requirements or research ethics requirements can disturb and disrupt established practices of data exchange, requiring knowledge networks and consortia to adapt to shifting or indeterminate applicable requirements. Rapid legal change, unsettled ambiguities in law, or conflicting obligations arising in different jurisdictions can exhaust the limited legal compliance resources of biomedical consortia, or deter the secondary use of research data for fear of legal non-compliance.

In the following sections, we examine the relationship between the legal and ethical rules applicable to information exchange, and the organizational and technological structure of data governance as practiced by biomedical research consortia. It is our contention that the relationship between legal and ethical rules, and consortium governance tools, is symbiotic. That is, approaches to self-governance, and instruments used to facilitate self-governance respond and adapt to the law, whilst also filling the gaps in the law and acting as a form of self-help to ensure normative interoperability despite considerable distinctions in applicable legal rules and bioethics norms across shifting geographies and jurisdictional boundaries. Normative interoperability here refers to the capacity of institutions to operate and to govern themselves in a coherent fashion across different legal regimes and legal requirements, in a manner that remains efficient and self-consistent.

## II. THE REGULATORY FOUNDATIONS OF DATA GOVERNANCE

Data protection legislation has experienced a significant revival of interest in recent years. Many countries have enacted new data protection laws or have amended existing data protection legislation to ensure its ongoing relevance in the face of technological change and rapid globalization. However, on the global level, there are no binding global international treaties devoted specifically to data processing and protection. General privacy considerations have been included in Art. 12 UDHR,^4^ which represents customary international law, with its privacy rules having been translated into Art. 17 ICcpR, ^5^ a universally binding international treaty, and also into Art. 8 ECHR,^6^ a regionally binding international treaty. Although judicial and scholarly interpretive work has led to them being understood as data protection rules, codification work in this area is still rare. The only binding treaty on an international but regional level is the Council of Europe’s Convention 108+, modernized and amended by Protocol CEST Nr. 223.^7^ Beyond that, today’s data protection laws are generally derived from the 1980 OECD Privacy Guidelines, renewed in 2013. The OECD Privacy Guidelines—a non-binding international instrument—represent the first concerted global effort to create common standards for the use of personal data and the protection of individual privacy.^8^ Referring to the understanding of data governance as a tool to provide normative interoperability in data processing across different contexts, international data protection rules offer a particular contribution to a converged governance in the data sharing landscape across different levels of regulation. Partly different from these rules are the EU’s data protection laws, as they form part of the individual legal regime of EU law. However, the EU itself is an international organization and as such an important actor in international data governance, besides contributing to normative interoperability through strong harmonization of its Member States’ (internal) legal framework. Apart from the examples of the international organizations’ rules, Council of Europe 7 (CoE) Organisation for Economic Co-Operation and Development (OECD) and EU, mandatory disclosure rules, data localization rules as well as rules on international data transfers are particular regulatory areas that will be emphasized. These areas of law determine the relation between different laws, or function as applicable laws and so influence normative interoperability in the international data processing landscape.

### A. The Council of Europe’s Modernized Convention 108 (2018)

The modernized Convention 108 for the Protection of Individuals with regard to Automatic Processing of Personal Data is strongly oriented towards the EU’s General Data Protection Regulation (GDPR). This is understandable, as all members of the EU are also members of the CoE and have significantly influenced the adaptation of the revised Convention to apply in a manner aligned with their obligations resulting from EU primary and secondary law. Nevertheless, the Convention establishes important rules, now integrated into public international law, including a definition of the main data protection principles, established by the OECD, differentiated individual rights of data subjects, data security measures, and rules on supervisory authorities as well as on international data transfers. Furthermore, it recognizes scientific research purposes as an individual legal basis for the processing of personal data.^9^ That is, Convention 108+ explicitly establishes that the processing of personal data for scientific research purposes, including further processing beyond its original purpose of collection, should be considered lawful so long as appropriate safeguards are utilized.^10^

Although almost all Member States of the CoE have signed the treaty, ratification is still not complete. Adherence by non-Member States opens up the potential scope of application of the treaty and could provide for its global effect. However, expectations in this respect must be kept low.^11^ Thus far, only eight countries that are not CoE Member States have ratified Convention 108.^12^ No non-Member State countries that are heavily engaged in the international exchange of data have ratified the Convention as of yet.

### B. The OECD Privacy Framework (2017)

The OECD Privacy Framework enshrines a number of privacy principles that have been subsequently mirrored in later data privacy and data protection laws around the world.

The principles are as follows:^13^

1. Collection Limitation
2. Data Quality
3. Purpose Specification
4. Use Limitation
5. Security Safeguards
6. Openness
7. Individual Participation
8. Accountability

### C. The Data Protection Directive (1995)

The first major data protection law with broad application and implementation measures is the Data Protection Directive (DPD) enacted by the European Union in 1995.^14^ Prior to the enactment of the DPD, individual rights in data generally consisted of limited individual rights to privacy, as enshrined in constitutional law and the law of civil liability.

The DPD introduced a number of innovations. Providing a full overview of the contents and implications of the DPD is outside the scope of this essay. However, certain concepts central to the DPD have been replicated in a number of other data protection and data privacy laws.^15^ Consequently, the DPD provides the foundation from which much of contemporary data privacy and data protection law is derived.

The DPD was implemented by the European Parliament in the form of a ‘Directive’, rather than a ‘Regulation’. European Union law recognizes both Directives and Regulations. Directives must be implemented into the local law of each EU Member State and introduce only a minimal binding standard. Conversely, Regulations are directly applicable in all Member States of the EU. As a result, the DPD was not a singular law applicable in each EU Member State. Rather, each individual EU Member State implemented an enabling law, sometimes adopting stricter approaches.^16^ Directives, in contrast to Regulations, function similar to general EU internal frameworks, which limits their capacity to harmonize the national law of EU Member States.

### D. The General Data Protection Regulation (2016)

The GDPR was adopted in April 2016 and implemented in May 2018. The GDPR is a Regulation, and this, in contrast to a Directive, finds immediate and direct application in all EU Member States. The GDPR includes a broader room for individual Member States to make their own rules than do other EU Regulations. The GDPR creates a heightened potential for individual Member States to make their own rules than do other EU Regulations. This room allows them to incorporate stricter, locally applicable changes to the general functioning of the GDPR. The predecessor of the GDPR, the DPD was adopted in the form of a ‘Directive’, rather than a ‘Regulation’. As a result, the DPD was not a singular law applicable in each EU Member State. Rather, each individual EU Member State implemented an enabling law, sometimes adopting stricter approaches.^17^ As such, the DPD constituted more an internal interoperability framework with only little harmonizing effect. However, the GDPR also functions in a number of ways as an internal framework subject to local implementation rather than as a singular harmonized body of EU law.

The territorial application of the GDPR is greater than the territorial application of the DPD. For instance, the personal data of individuals outside the European Union may be governed by the GDPR if the data are processed (ie held or utilized) by a controller established in the EU. The GDPR also applies to how goods and services are offered to individuals in the EU by entities outside the EU, and to how the behavior of individuals in the EU is monitored by entities outside the EU.^18^ In this case, the GDPR requires the entirety of its rules to be applied outside the EU^19^ in order to protect individuals inside the EU, even when non-EU parties process their data. This mandated focus on the protection of data subjects, rooted in primary EU law and the constitutional laws of Member States renders the imperative toward normative interoperability with the laws of non-EU jurisdictions subordinate to the protection of EU fundamental rights, and extends its scope of application outside of the territorial boundaries of the EU and EEA (for the application of transfer rules cf. sec. 8 below).

Other changes introduced by the GDPR include the codification of individuals rights previously established in jurisprudence, such as an explicit right to data erasure^20^ (the ‘right to be forgotten’)^21^ and the creation of new rights altogether, such as the right to data portability^22^ (ie the right of individuals to be provided with their data in an easily portable format).^23^ The GDPR also includes a number of other new features relating to the implementation of these new individual rights. An obligation to ensure ‘data protection by design and by default’ has been adopted.^24^ This obligation mandates controllers and processors using personal data to ensure data protection not only in the direct processing of personal data, but also in establishing the structure of the organization of the data and creating technological designs.^25^ In essence, this means that technical measures function as an operationalizing tool for the implementation of the fundamental right to data protection in its various facets, declaring technological solutions for privacy crucial for implementing and applying individual rights.

In addition, the GDPR’s rules on broad consent open up its data protection framework for communication with ethical standards in medical and health research by mandating adherence to recognized ethics standards when such consent is applied. A further opening towards governance is the possibility to create sector-specific laws based on codes of conduct, whereby normative interoperability is achieved through the involvement of various actors in creating those rules, providing room for recognition of non-legal behavioral norms that might steer data processing activities in certain research fields.

New administrative responsibilities have been adopted that require controllers to perform certain tasks prior to processing data. Such tasks include conducting a data protection impact assessment prior to using personal data if such use could pose a high risk to the rights and interests of the individuals concerned.^26^ Controllers are also required to maintain records concerning the personal data processed and the individuals it concerns.^27^ Risk assessment and documentation rules both serve to extend the applicability of these obligations beyond the brief period of data processing, extending the scope of application of data protection law over time.

### E. Data Protection Law in Other Countries

A number of other nations have recently adopted data privacy laws, or are in the process of adopting such legislation. Certain countries that implemented data privacy legislation a long time ago are amending their data privacy legislation or introducing novel data privacy laws.^28^

The first approach is sector-specific, as typified by the USA’s approach to data privacy. The USA has specialized laws such as the *Health Information Portability and Accountability Act* (HIPAA), which governs covered entities in the health sector,^29^ and the Federal Trade Commission (FTC) *Privacy of Consumer Financial Information Rule*.^30^

Numerous other jurisdictions have adopted an approach to data privacy law that lies at the intersection of the holistic European ‘data protection’ approach and the sector-specific USA ‘privacy’ approach. For example, hybrid legislative characteristics are common to data privacy laws adopted in jurisdictions such as Canada,^31^ Japan,^32^ and South Korea.^33^ Such hybrid legislation generally regulates data distinctly for each economic sector or across specified contexts of data use (eg private-sector legislation, public-sector legislation, and health-sector legislation). Certain kinds of information, which can include information used for journalistic purposes or information related to employment, are carved out of the ambit of data privacy statutes (eg on the understanding that such data should not be considered personal or private in nature). Specialized statutes are used to govern certain distinct contexts of data use. These latter features are reminiscent of the American approach to privacy legislation.

Yet, other features of such legislation are more akin to the European approach to data privacy. For instance, a justification in law (ie a lawful basis) must generally be demonstrated as a precondition to the use of personal data. Furthermore, many of the obligations enshrined in the international data protection codifications (eg Council of Europe, OECD, and the GDPR) are integrated within other countries’ data protection statutes, including requirements to use security safeguards to hold data safely, to remain accountable for the ongoing use of data, and to respect certain ongoing individual rights relative to personal data.

The structural similarities between European data protection and the national and sectoral data privacy laws of other countries are thus not coincidental. Such similarities arise partly because, as mentioned, the 1980 OECD privacy guidelines served as a common template for the creation of many national and regional data protection laws.^34^

These similarities also arise because other countries have intentionally legislated to ensure that their data privacy legislation is sufficiently similar to that of the European Union so as to be deemed ‘adequate’ by the European Commission. To reiterate, the European Commission can issue an adequacy decision in favor of a nation, a sub-national territory (eg a prefecture, province, or state), one or more specified economic sectors, or an international organization. Once such a finding is made, the transfer of personal data from the European Union to the recipient destination becomes significantly less burdensome from a legal compliance standpoint. Therefore, countries have ensured that their legislation is similar to the data protections laws of the EU/EEA to facilitate data sharing from the EU/EEA^35^ (cf. Section 8 ‘International Data Transfers’ below).

### F. Data Localization Laws

Another category of laws that are similar to, but conceptually distinct from, data privacy and data protection laws are data localization laws. Such legislation generally forbids the transfer of individuals’ personal data outside of the country in which the data were originally collected. Strict data localization laws forbid the storage, transfer, or use of personal data extraterritorially in most or all cases. Less onerous data localization laws impose significant preconditions on the international transfer of personal data, or limit such transfers to State-approved circumstances or legislatively specified use-cases (cf. Section 8 ‘International Data Transfers’ below).^36^ Furthermore, some jurisdictions have incorporated to their national data protection legislation default presumptions against the use or the movement of certain special categories of personal data, absent exceptional justification. Such categories of data often include biometric data, health data, and genetic data,^37^ or data that is generated in local health institutions.^38^ Numerous countries including China,^39^ Russia,^40^ and the Canadian province of British Columbia^41^ have adopted one form or another of data localization legislation.^42^ Even some EU countries, such as Germany, have imposed limitations on data processing in specified healthcare contexts. These limitations exercise a comparable function to data localization laws. The processing of personal data by a digital health application, as well as processing of personal data on behalf of a third party through such an application, may only take place in Germany, the EU Member States, in the EEA, in Switzerland, and in third countries for which an adequacy decision has been issued by the European Commission.^43^ Accordingly, appropriate safeguards pursuant to Art. 46 GDPR cannot operate as transfer mechanisms for such data processing. Furthermore, derogations such as the explicit consent of the data subject under Art. 49(1)(a) GDPR cannot legitimize a data transfer in this context. Minimum requirements that providers with parent companies in third countries without an adequacy decision must fulfil in order to be able to process personal data through such applications on behalf of the manufacturer take into account both technical measures in the form of encryption of all data controlled by the manufacturer, as well as organizational measures that provide a sufficient guarantee to prevent a data transfer outside of the scope of application of the GDPR to the parent company.^44^ However, insured persons may be physically present in a third country for which there is no adequacy decision. In such a case, health data may pass through a server in that country, in which case its law applies to the insured person and the processing is no longer the responsibility of the manufacturer. The offering of health apps via the online stores of providers in third countries that are not subject to an adequacy decision is possible if the login data are strictly separated from the health data, because the login data are processed for the purpose of downloading and updating the app, whereas the processing of health data serves other purposes.^45^

### G. Mandatory Disclosure Requirements

The third category of laws relevant to data governance is access to information laws, and other legislatively enshrined requirements to disclose data. Such laws include public-sector ‘access to information’ legislation, which requires government agencies to make the information they hold available to the public in anonymized format on request. Other examples thereof include the disclosure requirements imposed by medicines agencies (eg the European Medicines Agency and Health Canada), which require the public disclosure of clinical trial results relating to drugs and medical devices.^46^

Both access to information laws and clinical trial disclosure requirements generally necessitate the public disclosure of data, but mandate that personal data are not to be disclosed. Access to information legislation often provides a number of further justifications for the non-disclosure of data, including commercial interests, State interests, or the prohibitive impracticability of accessing and disclosing the desired data.

If data cannot be disclosed for reasons of confidentiality or privacy, both clinical trial disclosure policies and access to information legislation generally require the data to be de-identified and disclosed in an anonymized format.^47^

### H. International Data Transfers

Finally, some countries and supranational organizations, including the European Union, impose special limitations on the transfer of personal data to third countries. In this section, we describe certain limitations imposed by the law of European Union on outbound data transfers from the EU to third countries. The GDPR, as previously discussed, establishes that outbound data transfers need to respect certain legal requirements in order to uphold the level of protection if data are shared outside the EU or European Economic Area (EEA).

### I. Adequacy

The preferred mechanism for data transfer from the EU to third countries is a transfer performed in reliance on an adequacy decision (ie a determination by the European Commission that the destination of the transfer provides a comparable level of data protection as the European Union). The European Commission can declare the standard of data protection offered in third countries, sub-national territories, or by select international organizations to be ‘adequate’. Such a designation establishes that transfers of data to the recipient country, territory, or organization are presumed lawful, rather than requiring a distinct and exceptional admissibility. To date, only a small number of countries have benefited from an adequacy decision in their favor.^48^ These decisions generally benefit a specified country or territory, or—more recently^49^—apply in favor of the data that is governed by a specific law within a territory.

### J. Transfer Safeguards and Transfer Derogations

If no adequacy decision is available, the transfer must be performed on the basis of another safeguard established in the GDPR [eg standard contractual clauses approved by the European Commission, binding corporate rules (BCRs), or a code of conduct]. If no such transfer safeguard is available, certain exceptional derogations from the usual protection of EU law are available to transfer data, eg for important reasons of public interest or by relying on the explicit consent of the data subject.^50^

Certain EU court decisions have also affected the measures that must be taken to transfer data from the European Union to third countries. In *Schrems I*^51^ and *Schrems II*,^52^ the Court of Justice of the European Union (CJEU) considered the potential effect of international data transfer from the EU on the fundamental rights of data subjects in the EU.

It concluded that transfers from the EU to jurisdictions in which authorities perform mass surveillance on electronic communications (eg the USA) or those which access the electronic communications of individuals without sufficient due process could violate the fundamental rights and freedoms of data subjects in the EU.

Such interference by the authorities at the destination of the transfer with the fundamental rights of EU data subjects are beyond what is necessary and proportionate, in the pursuit of objectives recognized in EU law.^53^ This can also be the case in a number of other instances, for example if individuals concerned by State surveillance are not provided with sufficient notice thereof or are not provided with opportunities to exercise effective recourse to ensure the respect of their fundamental rights.^54^ European Union law therefore requires entities in the EU transferring data to other jurisdictions on a basis other than an adequacy decision to *assess* whether the law of the recipient country and the practices of its authorities are capable of ensuring that the fundamental rights of EU data subjects are respected.^55^*Schrems II* established that a data transfer performed on the basis of an adequacy decision according to art. 45 (1) GDPR, or on the basis of a transfer mechanism according to art. 46 GDPR, could be prohibited or suspended if it was not possible to guarantee respect for the fundamental rights of data subjects in the EU,^56^ as established in the jurisprudence of the CJEU^57^ and the European Court of Human Rights (ECtHR).^58^ The European Data Protection Board (EDPB) has formalized as the ‘European Essential Guarantees’ the minimum protections from surveillance that the law and the legal system of a recipient jurisdiction must provide for it to be possible to transfer data to such a jurisdiction whilst respecting the fundamental rights of data subjects in the EU.^59^

### K. Additional Measures to Protect the Fundamental Rights of EU Data Subjects

If the law or practice in the country of destination is not capable of ensuring respect for the fundamental rights of EU data subjects, additional measures must be implemented to further protect data prior to performing such a transfer.^60^

The EDPB considers that these could include coding data prior to transferring it to third countries, and retaining the ‘linkage log’ in the European Union or in another country considered adequate.^61^ This has important implications for the sharing of pseudonymised (coded) data, in that it provides a clear path to the international sharing of coded biomedical data in compliance with the GDPR. The EDPB also considers that the use of secure multi-party computation could in some instances also satisfy this requirement.^62^ Overall the EDPB considers that technical measures that actively prevent authorities from accessing the data of EU data subjects must be imposed to ensure the respect of the fundamental rights of EU data subjects, in instances where organizational measures or physical measures could be overcome by State authorities.

The transfer rules of the GDPR have often been evaluated as rules of applicable law^63^ that strengthen the extraterritorial effect of the Regulation. However, it needs to be emphasized that the latest developments resulting from the *Schrems II* judgment of the ECJ^64^ and the EDPB position on the role of supplementary measures to secure international data transfers clearly enforce the privacy-by-design principle of EU data protection law, as they assign technological tools the role of removing obstacles. This is true not just for conflicts under applicable law but also for secure normative interoperability in the sense of communication between applicable rules in a broader sense, including non-legal norms. In addition, particular consideration must be given to codes of conduct that can also function as a tool for the admissibility of international transfers and play an important role in coordinating non-legal norms in cross-border matters as well.^65^

## III. DATA GOVERNANCE AND BIOETHICS

Data protection law imposes procedural requirements on the use of personal data, and grants individuals concerned by personal data substantive rights in their data. However, a second objective of data protection legislation is to foster the use of data for the purposes of economic development and scientific innovation. Indeed, data protection is an emergent regulatory framework subject to a number of practical controversies, and elements that require further clarification through regulatory guidance, and community or industry guidance (ie through approved codes of conduct and BCRs). Furthermore, soft law guidance becomes particularly relevant when rights and interests concerning data protection need to be balanced against interests arising from data processing that are also rooted in human and fundamental rights such as the right to research freedom and the right to benefit from scientific development. In addition, bioethical principles and values can guide the data protection compliance efforts of health consortia, merging relevant rules into a governance framework. Such guidance can be directly relevant to long-term decisions made by health consortia in balancing their many duties stemming from ethics, law, and public policy. This way, interpretive guidance and the codification of ethical standards can foster sector-specific normative interoperability between different normative systems mandating rules for data processing.

### A. Biomedical and Research Ethics Guidance

#### 1. Principles of Biomedical Ethics

Bioethics practices and research ethics guidance provide a number of conceptual approaches that are useful in considering the individual and collective dimensions of personal data use and personal data sharing. The most notable sources of ethics guidance in biomedical research are the 1947 Nuremberg Code, the 1964 Declaration of Helsinki (revised 2013),^66^ and the International Ethical Guidelines for Health-related Research Involving Humans (CIOMS/World Medical Association (WMA) 2016).^67^ There also exists guidance specific to international clinical trials (eg the ICH Harmonized Guideline).^68^ Such international bioethics guidance has placed much emphasis on interventionist research. However, there is a growing body of international research ethics guidance that is directed to database science, longitudinal studies, and biobanks (eg the 2016 Declaration of Taipei of the WMA).^69^

Traditional medical ethics literature introduced the four principles of autonomy, beneficence, non-maleficence, and justice in guiding ethical decision-making.^70^ These principles have influenced the development of the medical ethics guidance and research ethics guidance concerning data as well. No hierarchy exists between the principles discussed—all must be considered both contextually and holistically.^71^

#### 2. Equity in International Health Research

Heightened research participation by scientists in countries historically excluded from research participation is as a critical driver of increased health equity. Health research is becoming increasingly data-intensive, and participation therein is therefore contingent on access to large biomedical datasets.^72^ Further, access to the significant technological infrastructure to format, analyze, store, and disseminate big biomedical data is a further prerequisite of full-fledged participation in data-driven health research.^73^

In light of these imperatives, there are strong policy justifications for ensuring that data protection legislation does not impede the participation of researchers from developing economies in health research, nor frustrate scientific research that benefits societal groups that have historically been deprived of benefit from scientific research.^74^

### B. Data Research Ethics

The foregoing medical ethics principles have greatly influenced international approaches applicable to research data governance, and the longitudinal preservation of human biological materials and data. Several common thematic approaches have emerged in the last decades: scientific commitment to open data sharing, international commitment to shared benefits from biomedical data, and international standards bodies for biomedical and genomic data.

#### 1. Scientific Commitment to Open Data Sharing

Representative bodies in the scientific community, especially scientists working in the areas of genetics, genomics, and bioinformatics, have adopted principles and statements that affirm the scientific community’s commitment to the rapid and open release of genetic and genomic data.^75^ The genetics and genomics communities, including numerous private-sector partners, have an established culture of publicly releasing data by default.^76^

This practice was instilled at the advent of the Human Genome Project (HGP), led by the Human Genome Organization (HUGO), principally through the advocacy of the scientific community and its commitment to HUGO Ethics Statements with a particular emphasis on engagement and benefit sharing. This commitment to the open sharing of genomic data was affirmed in the Bermuda Principles (1996),^77^ the Fort Lauderdale Agreement (2003),^78^ and the Toronto Statement (2009),^79^ amongst others. These statements reiterate the scientific practice of sharing genetic and biomedical data openly and widely. The general commitment to openness is counterbalanced against certain other values, such as research participant privacy and the right of scientists to attribution and publication of their findings. Consequently, contemporary policy statements on the sharing of genetic and biomedical data often accept the implementation of certain access controls, such as managed/controlled access or embargo periods requiring secondary data users to accord priority of publication to the original creators of the dataset.^80^ The UNESCO Draft Recommendation on Open Science further promotes open access to data, made conditional on appropriate data governance mechanisms, as one of its central pillars.^81^ Novel mechanisms for the advancement of open science are also proposed. The approaches articulated in the UNESCO Draft Recommendation include public–private partnerships to promote industry engagement in open science initiatives, and the development of laws and national policies that enable open science. Other actions proposed therein include the open licensing of research outputs, and increased State investment in technological infrastructure, such as internet access, computational resources, and non-commercial information repositories.^82^

#### 2. International Commitment to Shared Benefit from Biomedical Data

International commitments to the open sharing of biomedical and genetic data are also reflected in statements establishing balancing of the human right to participate in research and benefit from its products, individual confidentiality interests, and ethical interests in the welfare of research participants. Such instruments include the, Universal Declaration on the Human Genome and Human Rights (UNESCO, 1997),^83^ the International Declaration on Human Genetic Data (UNESCO, 2003),^84^ and the Universal Declaration on Bioethics and Human Rights (UNESCO, 2005).^85^ Other relevant policy statements include the Guidelines on Human Biobanks and Genetic Databases (OECD, 2009)^86^ and the Recommendation on Research on Biological Materials of Human Origin (Council of Europe, 2016).^87^

The articulation of anticipated rights and responsibilities differs in each of these documents. However, certain general commitments are articulated throughout. Three such commitments include the following:

First, there is a common agreement on the need for international collaboration and cooperation in the analysis and utilization of biomedical and genetic data. It is understood that such commitment should be actualized in the structure of domestic law that governs domestic and international data flows.^88^ It is also understood that such sharing should translate into the liberal utilization of data for diagnostic purposes, research purposes, and health-sector capacity-building purposes in the form of benefit sharing.^89^ Ensuring the equitable participation and access of low and middle income countries to data and data processing infrastructures is another recognized international priority.^90^

Second, a number of other data collection and communication practices have been agreed on as an extension to binding data processing rules, contextualizing them in the medical research and healthcare settings. These practices relate to the gathering of informed consent,^91^ the return of research results and incidental findings,^92^ and the secondary use of research data and biomaterials.^93^ Such pronouncements establish international comity relative to ethics oversight and longitudinal data governance practices. These practices generally entail respect for participant autonomy both to participate in research and the right to choose *not* to be informed of their genetic information. These practices also establish a general right to the free circulation of genetic and biomedical data so long as such sharing and use occurs in compliance with best ethical practice (eg oversight, involvement of the participants in decision-making, review of research protocols, etc.).^94^

Third, accepted definitions for gradations of biomedical data identifiability and the permissible uses of data according to its identifiability are enshrined in such data ethics guidance.^95^ It is generally understood that individuals shall have a great degree of control over the acceptable uses of their genetic data in identifiable form (ie in direct association with their direct identifiers), absent certain limitations thereto established in domestic law (eg for select direct healthcare provision purposes and criminal law purposes, or for scientific research purposes in accordance with domestic law).^96^ Furthermore, secondary uses of data in anonymized or coded form (ie all direct identifiers are removed and replaced with an alphanumeric code) are often considered to be acceptable.^97^ Individual control of the permissible uses of data,^98^ and the requirement to subject the use thereof to rigorous ethics oversight, are more limited where the data are irreversibly anonymized (ie irreversibly de-linked).^99^

#### 3. International Standards Bodies for Biomedical and Genomic Data

Another source that translates general concepts of biomedical ethics into practical application is the guidance of international standards bodies that create technical and organizational policies, tools, and standards for the governance of biomedical and genomic data. Prominent examples thereof are the Global Alliance for Genomics and Health (GA4GH), H3 Africa, and the Research Data Alliance (RDA). These bodies should not be confused with standardizing bodies such as the International Standards Organization (ISO) or the International Electrotechnical Commission (IEC). The latter categories of bodies foster the adoption and maintenance of specific technical standards throughout the world, to which legislators and regulators often refer in developing domestic technical standards.^100^ Conversely, most standards bodies acting in the realm of bioethics create template policy documents and technical tools for future use by concerned stakeholders, rather than attempting to create harmonized and uniform practices, internationally, within a concerned sphere of activities. Nonetheless, the documents and tools created by international standards bodies in bioethics have fostered compatibility and commonalities in the practices and procedures of international biomedical research consortia.

The GA4GH was founded in a human rights framework, namely ‘The Framework for Responsible Sharing of Genomic and Health-Related Data’.^101^ The GA4GH has issued a number of guidance documents and tools to facilitate compliance with ethical and regulatory data governance requirements, and to foster compatibility of data stewardship practices amongst multiple institutions.^102^ Guidance policies include: the Consent Policy,^103^ the Copyright Policy,^104^ the Data Privacy and Security Policy,^105^ the Accountability Policy,^106^ and the Ethics Review Recognition Policy.^107^ There are also toolkits such as the Genomic Data Toolkit, which centralizes the use of metadata standards, ontologies, and e-consent approaches to facilitate researcher understanding of and respect for informed consent requirements, and ethical and legal data use conditions.^108^ The Regulatory and Ethics Toolkit, includes tools such as generic consent clauses, guidance for holding data secure, and for public engagement.^109^ Last, its Data Security Toolkit provides technical and cryptographic methodologies to ensure that data are held secure from a technical standpoint.^110^

Human Heredity and Health in Africa (H3 Africa) is a research consortium established in 2010, in a collaboration between the African Society of Human Genetics, the African Academy of Sciences, the National Institutes of Health (NIH), and the Wellcome Trust.^111^ The H3 Africa consortium has issued an Ethics and Governance Framework for Best Practice in Genomic Research and Biobanking in Africa^112^ and comprehensive bioethics guidance, as well as template documentation and guidance pertinent to informed consent and community engagement,^113^ the return of individual genomic findings,^114^ and a publication policy prioritizing the involvement of local scientists.^115^ The Governance Framework and other documents issued by the H3 Africa consortium place particular emphasis on community involvement, the avoidance of group harm and stigma, and equitable benefit sharing, among other values.^116^

The RDA is an organization dedicated to the open and interoperable sharing of research data amongst scientists worldwide. The organization was founded through the integration of data sharing initiatives of Europe’s Data Access Interoperability Task Force (DATIF), the US National Science Foundation (NSF), and the US National Institutes of Standards and Technologies (NIST).^117^ The RDA operates through the creation of Working Groups (WGs) that operate for 12–18 months and are mandated to develop practical deliverables related to a specific policy issue or technical problem related to data interoperability and data sharing.^118^ The RDA has released a large number of deliverables through this mechanism, including academic publications, surveys, recommendations and policies for the conduct of research, and software tools.^119^

A table detailing the functioning of the GA4GH, the RDA, and the H3 Africa consortium, is provided in Appendix 1 (Table A1). The table also provides direct links to the numerous policy documents and governance document templates of each of the GA4GH, the RDA, and H3 Africa.

The adoption of these tools by other consortia serves to foster interoperability between different normative standards for data processing and sharing. Having addressed international legal and policy instruments that crystallize the requirements of international bioethics, and the tools available, we now outline how data governance considerations can be completed by normative interoperability between the regulatory foundations and bioethical guidance related to data-centered biomedical research for health researchers, research institutions, and bioethics specialists.

### C. Normative Interoperability

Normative interoperability posits that distinct ethical and legal regimes should be able to interact meaningfully despite a lack of overt substantive and structural harmonization. Just as computer programs can interact meaningfully despite having been created in different programming languages, organizations should be able to engage in collaborative projects of data use despite being beholden to distinct research ethics requirements and disparate legal norms. In practice, ensuring normative interoperability refers to the actions of regulators or of regulated parties to create stable institutional and organizational practices between collaborators that are subject to distinct legal requirements or statutory frameworks.

For regulators, this can mean creating laws that grant regulated parties sufficient discretion in deciding the methods to use in achieving compliance, so as to ensure that distinct legal regimes do not create significant challenges arising from the conflict of laws. For regulated parties, this can mean designing common institutional and organizational arrangements that translate the distinct legal requirements applicable to each collaborator into a common operational strategy shared across a network of collaborating parties. National legislators and international rules can prepare the ground for normative interoperability.

First, international obligations and harmonization, as well as interpretive rules that reconcile potential conflicts between simultaneously applicable laws can contribute to overall data governance as a response to divergent data protection rules in different legal systems. That is to say, national legislators can facilitate normative interoperability by proactively working toward the harmonization of the law through coordinated efforts to adopt the same requirements across multiple national laws, or in instruments of public international law.^120^

Second, communication between divergent data protection regulations can be fostered by interpreting data protection rules in light of international bioethics norms applicable in the health research setting, thereby ensuring comprehensive adherence to shared norms related to biomedical research. In practice, this could be achieved if national data protection regulators issued regulatory guidance stipulating that biomedical researchers should defer to local or international research ethics requirements to interpret their data protection obligations, in instances of legislative ambiguity. This contributes to achieving normative interoperability by encouraging regulated entities to use established bioethics norms with longstanding histories of interpretation, and relative stability across different countries, to interpret their respective data protection obligations. Normative interoperability is thus enhanced between binding research ethics guidance and data protection law, and between the data protection norms of distinct jurisdictions.

Third, instruments of co-regulation, such as codes of conduct or approved technical measures, can be used to obtain regulatory approval of the legal compliance of specific organizational or technological mechanism that health-sector experts propose as best practices for discharging data protection obligations. That is, health-sector experts could propose common institutional practices, organizational structures, and technological mechanisms as tools for ensuring data protection compliance in distinct jurisdictions. These experts could then petition their local data protection authorities to approve these as established methods of ensuring compliance; this ensures the interoperability of compliance methods in the health sector internationally, despite the heterogeneity of local statutes. The GDPR, for example, includes numerous mechanisms designed to facilitate sectoral coordination of compliance methods, such as BCRs, certification marks, and codes of conduct.^121^ Efforts toward achieving normative interoperability attempt to foster the development of stable organizational arrangements despite heterogeneity or overlap in the legal, ethical, and institutional rules applicable to distinct actors engaged in the creation of centralized or decentralized infrastructures for data exchange.

Indeed, the majority of efforts at legal and general normative interoperability are derived not from public-sector or regulatory interventions. Rather, many innovations that create normative interoperability are the product of either international academic research endeavors or of the private sector. Such innovations include, for example, the use of negotiated contractual agreements between multiple research institutions to ensure that shared data conforms to the legal requirements applicable to each partner,^122^ the ethics single review of multisite research (US Federal Regulations, Common Rule 2019), and the use of compatible intellectual property licensing schemes across multiple collaborating research institutions.^123^

To reiterate, it is notable that the law itself fosters private actors’ initiatives to create greater legal and normative interoperability. This occurs where the law leaves aspects of its functioning to be devised by private actors. In the context of data privacy and data protection legislation, examples of such ‘outsourcing’ include the creation of sectoral codes of conduct or BCRs.^124^ Other examples include the determination of the appropriate security safeguards to be used to ensure that data are held and shared according to an appropriate standard of privacy and security.^125^ Having provided an overview of the conceptual relationship between existing health data governance approaches, data privacy, and data protection legislation in the context of normative interoperability, we now discuss specific data governance methods derived from the practice of international biomedical research consortia.

Other tools for increasing the interoperability of data across competing regulatory spheres include the application of codes denoting ethico-legal permissions to datasets as a form of metadata (eg using methodologies such as GA4GH’s ADA-M, Consent Codes, and the Data Use Ontology).^126^ These methods foster interoperability by ensuring that normative requirements imposed in the data’s jurisdiction of origin follow the data as it is transferred to other countries with different norms, while providing for their usability in different regulatory landscapes. Tangibly, these tools act to ‘tag’ data with markers describing either the jurisdictional provenance of the data, the substantive legal requirements applicable thereto, or the contents of local data governance policies applicable to such data, to help downstream users understand the permissions and restrictions inherent in the use thereof.

## IV. ORGANISATIONAL APPROACHES TO DATA GOVERNANCE

Organizational approaches to data governance can be beneficial to health researchers in discharging their ethical and legal duties to safeguard health data. These methods include core consent elements, consent filters, and consent tools; access controls and access federation; public communication and ongoing dialogue; expert governance bodies; and the implementation of contracts and policies. Data protection duties relating to ongoing accountability, transparency and openness, notification and individual participation, as well as data quality and data accuracy, are also best discharged in reliance on a robust organizational structure.^127^

### A. Core Consent Elements, Consent Filters, and Consent Tools

Health consortia have often relied on a combination of decentralized institutions contributing data to a centrally managed research infrastructure that is managed by a singular institution. Consortia that have adopted such a structure include the Canadian Partnership for Tomorrow’s Health (CanPATH),^128^ the ICGC, ^129^ and the MSSNG Database for Autism Researchers.^130^

To ensure respect for both local ethics practices and the regulatory requirements of contributing institutions, whilst also ensuring the harmonious administration of the entire database, consent tools are primary. Core Consent Elements constitute a series of mandatory minimum ethico-legal permissions for data collection and use that must be obtained by prospective data contributors. Establishing such a list of prerequisites to data contribution ensures that the datasets present in the database can be used for common purposes, without data custodians or downstream data users being required to manually assess the permissions inherent in each dataset relative to the intended secondary uses thereof. Such an approach is less rigid than mandating each data contributor to obtain *identical* permissions in data or to utilize the same consent materials across each participating research site.^131^

Template consent materials common to a health consortium can further help local research institutions and researchers ensure that their research consent practices and research consent forms reflect the core consent elements required to contribute data to the consortium. Each contributing research institution can also meaningfully assess the compliance of their legacy data with the core consent elements of a consortium, by using a retrospective consent filter.

Such a filter provides a series of questions for researchers hoping to determine if the ethico-legal use conditions applicable to previously collected (legacy) datasets are compatible with the core consent elements required to submit data to the consortium.^132^ For example, the HCA, the ICGC, and Canada’s National COVID-19 Immunity Task Force (CITF) have implemented core consent elements, template consent materials tailored to specific populations and sample collection contexts, and a retrospective consent filter to ensure the interoperability of its contributed data whilst also allowing local institutions to tailor their consent process to local norms.

In practice, the utilization of these tools is performed as follows. A consortium first establishes core consent elements that reflect the anticipated minimum use permissions that downstream data users will require to make plentiful use of the data. These minimum permissions are translated into template consent clauses or a template consent form that are made available to prospective users on the consortium’s central webpage.

Researchers that are prospectively collecting biosamples and/or data for the purpose of depositing it in the central consortium database can use the list of core consent elements, and the template clauses or template form, to create informed consent materials that are aligned both with the minimum permissions required to contribute data to the research consortium, and with the local requirements that are applicable to the contributing researcher due to their local law, research ethics guidance, or institutional policies and practice.

Second, the consortium also develops a retrospective consent filter. This is a flowchart or guidance tool that is directed to researchers that intend to contribute pre-existing data to the consortium, without designing their informed consent materials for this explicit purpose. This could be the case for legacy datasets generated prior to the creation of the consortium, for datasets generated for an external research project and subsequently selected for contribution to the consortium, or for datasets to which no informed consent to research participation is applicable (eg clinical data or data derived from leftover clinical tissues). The retrospective consent filter is used to determine if the applicable informed consents to research participation, and other applicable institutional, ethical, and legal authorizations applicable to the data are sufficient to enable data contribution to the central consortium (ie if those permissions are aligned with the core consent elements of the consortium).

If this retrospective assessment demonstrates that the permissions applicable to the data are insufficient to enable the contribution thereof to the consortium, such a filter further proposes potential avenues to obtaining the permissions necessary to perform data contribution. These often include performing the de-identification of data to render it anonymized, re-contacting research participants to obtain a suitable consent, or obtaining an ethics waiver of informed consent from the relevant Research Ethics Board (REB).^133^

A table describing, and providing links to, the consent-related policy documents of numerous consortia is included in Appendix 2 (Table A2). These documents include the template informed consent materials, the minimum core consent elements, and the retrospective consent filters of numerous biomedical research consortia including the HCA, the ICGC, Canada’s national CITF, and the Personal Genome Project (PGP).

### B. Access Controls and Access Federation

Biomedical consortia often use data access controls and federated data access models to hold different categories of data with different standards of human oversight and security. For instance, it is common to hold anonymized individual data and aggregated data in open access, which is available to all. Individual-level coded data are usually held in controlled or regulated/managed access, which requires researchers interested in accessing such data to apply for access to such data and to demonstrate the capability to adhere to privacy and governance commitments relative thereto.

Registered access is a median tier of data governance, which requires individuals to create an account, be approved as a bona fide researcher, and enter into certain commitments prior to accessing data. This latter mechanism is usually applied to data that, while not highly sensitive, could pose potential confidentiality, privacy, or intellectual property risks if left in entirely open access.^134^ The use of a registration requirement can safeguard against risks arising from data mining or data scraping (eg automated re-identification attacks or data scraping for the purposes of integration to a competing database), without diminishing the accessibility of the data for bona fide scientific research purposes.

Last, it bears mentioning that certain consortia have implemented federated technologies^135^ that enable the joint analysis of decentralized datasets. This enables researchers to reap the benefits of pooled data analysis without incurring the data protection and data privacy risks inherent in sharing the underlying personal data used to perform such analysis. However, this should not be considered a catch-all solution to the tension between data protection requirements and the societal interest in access to rich health-related data for research purposes. Reliance on federated data analysis techniques can be technologically burdensome, and can place prohibitive limitations on the categories of analysis that can be performed. Federated analysis often requires the duplication of technological infrastructure at each participating node, rather than in a singular central node, increasing infrastructure costs. Further, it can create difficulties in ensuring the replicability of research and in aligning the technical format of disparate datasets, because the participants in a data analysis cannot analyze or manipulate the concerned datasets.^136^

A table detailing the access control models of numerous biomedical research consortia has been provided in Appendix 3 (Table A3). These include models of open access, registered access, and controlled access consortia, as well as certain hybrid models that incorporate multiple access tiers.

### C. Public Communication and Ongoing Dialogue

Health consortia can ensure ongoing transparency toward research participants, members of the public, and contributing research institutions using a combination of public-facing materials and direct communications with specialized personnel. The following mechanisms can be implemented to ensure that researchers contributing data, research participants, secondary users of consortium data, and other consortium stakeholders can access information relevant to the data governance practices of the consortium:

First, the use of internal working groups dedicated to ensuring cohesive data governance throughout a consortium can be helpful in ensuring that good data governance practices are adopted across the lifecycle of a consortium and throughout its different dimensions (eg ethico-legal, scientific, technical, etc.). Data governance and data privacy working groups should be composed of members specialized in bioethics, computer science, the life sciences, and the law. The determination of data identifiability, the integration of privacy-enhancing technologies (PETs) to data repositories, and the adoption of ongoing safeguards including contractual agreements and auditable systems require joint expertise across the many domains.^137^

Second, the use of a help desk or a specialized representative tasked with responding to queries relating to data governance and research ethics can be helpful to a consortium. Such personnel can assist contributing researchers in ensuring that data are collected according to the technical and ethico-legal requirements required to contribute data to the consortium. Further, a help desk or specialized representative can assist downstream data users in understanding the ethico-legal requirements imposed by a consortium and ensuring compliance therewith.

Consortia can also benefit from establishing who will be responsible for responding to the queries of research participants that originally contributed data. Often, the principal investigators of contributing studies, and their research institutions, will be responsible for responding to such queries. Such a structure is adopted as the main consortium does not retain the direct identifiers of the research participants and thus are poorly placed to respond to queries regarding their data. Furthermore, the principal investigator and the research institution have a pre-existing relationship with research participants, which facilitates communication between the research participant and the researchers/research institution. The researchers can then communicate with the personnel of the consortium to request that any necessary information be provided or any necessary acts be performed (eg to follow-up on data access requests, data destruction or withdrawal requests, etc.).^138^

Third, a central webpage can be used to inform the public about the research projects that have used a consortium’s data. This can help research participants having contributed data to the consortium to more easily remain aware of the ongoing uses of their data. The lay summaries, scientific abstracts, or primary texts of the consortium’s research publications can also be made available on such a webpage.^139^

### D. Expert Governance Bodies

Health consortia often use expert governance bodies to ensure that ongoing use of their data minimizes the risks described above. Risks addressed by such governance bodies include the potential for research participant re-identification to occur, for unqualified or malicious parties to gain access to health data, or for secondary uses of health data not to constitute legitimate scientific research.

Different consortia use alternate kinds of expert governance bodies. Such governance bodies can include high-level steering committees, which are responsible for overall consortium governance and design, or smaller and more specialized governance bodies responsible for administering access to sensitive datasets according to pre-established criteria. The latter bodies are oftentimes structured as Data Access Committees (DACs) or Data Access Compliance Offices (DACOs).^140^ Certain such governance bodies ensure that their membership reflects topic-specific scientific, ethical, and legal expertise to inform the assessment of the scientific practicability and the privacy risks inherent in a proposed research project that would require access to data that is subject to their stewardship. This can warrant the selection of scientific members from disciplines relevant to the concerned bioresource, to ensure that these members are well-equipped to understand the scientific contents of the data access requests that are submitted for their review (eg epidemiologists for public health databases, bioinformaticians or genetic researchers for genomic data resources). The incorporation of representatives from the communities that the operation of the bioresource most affects to governance bodies can also prove advisable, especially where the concerned bioresource intends to steward data belonging to communities that are traditionally excluded from research participation, or excluded from the benefits of scientific innovation.

Governance bodies are distinct from the aforementioned working groups, as whilst working groups are generally responsible for assessing potential risks and proposing solutions thereto, governance bodies are responsible for practical decision-making within a consortium.

For instance, a working group might determine that the disclosure of a certain number of single-nucleotide polymorphisms (SNPs) relating to an individual creates a re-identification risk whereas the disclosure of a smaller number of SNPs does not. A steering committee might therefore be responsible for implementing a policy limiting external access to the data to the threshold of SNPs established. A DAC or DACO would then be responsible for ensuring that external parties accessing the data made meaningful commitments to respect the access policies of the consortium.^141^ Details of the different categories of governance bodies that select real-world consortia have implemented are provided in Appendix 4 (Table A4).

### E. Contracts and Policies

The use of contracts and policies can establish expectations regarding the ongoing use of data between the consortium and other stakeholders including data contributors and downstream data users. Such contracts include data transfer agreements (DTAs) and material transfer agreements (MTAs).

Creating binding contracts for data contribution and data use that are interoperable can ensure that the obligations and expectations of data contributors are seamlessly communicated to the future users of consortium data. This despite the data contributors and data users never engaging in direct interaction. This structure ensures the legal interoperability of the use conditions applicable to data contributed to a consortium.^142^

A second category of useful policies are terms-of-use, terms-of-service, attribution policies, publication policies, and data quality policies. Such instruments are generally non-binding and can establish common expectations and common practices among participants in a health consortium. The scientific research community has a long-established practice of using both formal and informal sanctions to respond to the breach of such community expectations.^143^ Additionally, through labor-law measures, they can become binding. Formal sanctions imposed through binding contracts include the removal of non-compliant data from a platform, the denial of continued access to a platform’s data portal, litigation, or the loss of future research funding. Informal sanctions arising from the breach of policies and contracts, whether binding or not, include reputational harm or the loss of trust in a researcher or research institution.^144^ Appendix 5 (Table A5) contains a thorough list of the different categories of consortium policies and consortium agreements or template agreements that biomedical research consortia often utilize to facilitate the practice of data governance.

## V. PRIVACY-ENHANCHING TECHNOLOGIES

In the previous section, we addressed the capacity of organizational structures to ensure that the longitudinal preservation and sharing of scientific data respects normative best practices. It is critical to also consider the role of novel technologies in mitigating real or perceived conflicts between heightened data utility and the privacy interests of research participants and their communities.

Technological approaches to data governance attempt to facilitate compliance with data protection legislation, and to offer individuals demonstrable guarantees of good data governance. This is achieved in minimizing trade-offs between privacy preservation and increased data use. Generally, PETs include three categories of innovation, as established in the following taxonomy first introduced by Ira S. Rubinstein.^145^

The most thorough, referred to as ‘substitute privacy-enhancing technologies’,^146^ facilitate privacy compliance in performing data processing operations without the use of personal data (ie in ensuring data anonymization).

The second most thorough, referred to as ‘complementary privacy-preserving technologies’,^147^ function in collecting individual-level data but masking the identities of such individuals to the parties using the information. In the health sector, such approaches might include ‘double-coding’ data to ensure that the data, aggregate-level data querying platforms that add noise to results to preclude individuals from being re-identified from the search results returned, or the aforementioned federated approaches to performing data analysis across multiple decentralized institutions.

Last, there are also ‘complementary privacy-friendly technologies’.^148^ Such privacy-friendly technologies do not modify the identifiability of the data used. Instead, such technologies serve to facilitate compliance with other data governance requirements. This can include the automated creation of audit logs and records of personal data inventories, the implementation of dynamic user consent mechanisms, or the use of security safeguards such as network segregation or encryption. Together, all three families of technologies are collectively referred to as PETs.

Data protection laws often require ‘privacy by design’ or ‘data protection by design and by default’ to be implemented.^149^ Such obligations are sometimes formalized as an explicit obligation (eg the EU), or else carried across through more general legislative requirements to ensure that ‘technical and organizational’ approaches to data security are adopted.^150^ Furthermore, the use of PETs could prove essential in complying with legislative requirements to implement security safeguards and safeguards for international transfers enshrined in data protection legislation.

Privacy-enhancing technologies could be used as safeguards to ensure the legal compliance of international data transfers, or to ensure that data are held to an appropriate standard of security according to domestic data privacy law. However, as such a measure, these safeguards will need to respond to the actual rights and interests defined in the health-data context and at the consortium level.

### A. Differential Privacy

Differential privacy is a technical privacy guarantee that ensures that aggregate or summary-level data cannot reveal the data of the individual records from which it was generated. This is achieved in ensuring that the results of queries at the aggregate level are slightly skewed, such that the results of a single record do not sufficiently alter the results of aggregate-level queries to risk individual re-identification.^151^ For instance, if a single outlier record would significantly alter the average returned from a group-level query, that record can be excluded from the calculation of average results, or noise can be added to the calculation of average results. This is done to prevent the use of the average results returned to infer values related to specific records in the dataset, or to determine the inclusion of specific records in the dataset.

Differential privacy can be implemented at the dataset level for static data releases, in translating the raw data into synthetic data through the addition of noise, ideally in such a way as to preserve the statistical relationships between variables that are of research interest arising in the raw datasets concerned. Differential privacy can also be implemented in deriving aggregate information from record-level data, in adding noise to the aggregate data so as to ward the data against re-identification attacks. Last, differential privacy can be implemented to a search engine or query platform, to ensure that the queries made cannot be targeted to reveal information about the underlying records that could enable the re-identification of the individuals concerned.

Formally speaking, differential privacy stands for the proposition that the inclusion or exclusion of a single record from a dataset should not change the results of an aggregation or query directed to the dataset; noise is therefore added to the results generated to change the results obtained such that a single record’s data does appreciably alter the results of the aggregation process or query. However, the challenge inherent in implementing differential privacy is that a workable balance of privacy-preservation and data utility can be difficult to achieve. Technical solutions to this issue have included formalizing the amount of information leakage that a differential privacy method permits, in comparison to the reduction of data utility. The former process can entail introducing a privacy budget to a query platform, either at the platform level or on a per-user basis, that restricts access platform-wide to the data deriving from a specified record, or restricts a specified user’s access to the platform, once a specified threshold of information deriving from a single record has been revealed through the use of the platform.^152^

### B. Genomic Beacon Systems

The use of genomic beacon systems has been adopted to allow for the wholly open (ie open access) querying of variant-level information from among aggregate records concerning specified genomic records. The use of a genomic beacon system reconciles values of privacy and data utility in making the existence of genomic information known at the variant level, but in masking or altering the results of queries that are formulated in a strategic fashion to reveal genetic information about a specific individual.^153^

### C. Automated Data Minimization

One potential technical approach to reducing the potential for long-term data storage to create a heightened risk to individual privacy is to automate the deletion or anonymization of records using algorithms. This can be done at the point of data ingestion in automatically removing direct identifiers such as names and home addresses from ingested data. This can also be done on an ongoing basis, in automating the removal of data from datasets after a set period of time has elapsed or if criteria are met that demonstrate that the data are no longer of utility to the consortium.^154^

## VI. CONCLUSION

Data protection legislation is undergoing rapid evolution, refinement, and proliferation. Similarly, data governance solutions in the biomedical research sector are being developed and implemented at an equally rapid pace. Research consortia can act as standard-bearers for the alignment of regulatory, ethics and technical approaches to best-practice data governance. Future consortia could use the proposals described herein to guide the implementation of organizational, technical, and physical approaches to data governance. The approaches summarized in this landscape are instrumental in ensuring that research datasets produced for wide dissemination are secure and yet sufficiently rich to be of scientific utility while representative of the different population groups that contributed.

The ethico-legal governance measures of international consortia are established to navigate between legal and ethical provisions changing in time and space. Even though these measures often stem from private actors or self-assembled consortia, they provide interoperability and thus communication between different normative regimes. This communication is essential to allow international data sharing. Ethico-legal data governance measures therefore foster convergence between bioethics norms and data protection law, creating a common culture of data sharing in combination with appropriate safeguards and oversight. These governance practices encourage the standardization of biomedical data stewardship practices despite the lack of harmonization amongst national laws. Perhaps in the future, national legislatures and regulatory bodies could further enable the legal interoperability of health and biomedical data exchange by recognizing, explicitly or tacitly, the governance measures that biomedical research consortia already espouse as legal compliance best practices.

## ACKNOWLEDGMENTS

AB and BMK are funded by The Chan Zuckerberg Initiative (CZI); The Helmsley Charitable Trust; and the Klarman Family Foundation.

FMG is funded by the German Federal Ministry of Education and Research, Project TrustDNA (Grant Number: 16DTM108A)

## CONFLICT OF INTEREST

AB and BMK have no conflict of interest to declare. FMG is a member of the European Group on Ethics in Science and New Technologies. This paper is written in a purely private capacity and the views expressed here cannot be attributed to anyone other than the authors.

## Footnotes

1 See: Jane Kaye, Catherine Heeney, Naomi Hawkins, Jantina de Vries, and Paula Boddington, *Data Sharing in Genomics—Re-shaping Scientific Practice*. 10 Nat. Rev. Genet. vol. 5, 2009, DOI: 10.1038/nrg2573. PMID: 19308065; PMCID: PMC2672783.

2 See: Lincoln Stein, Bartha M. Knoppers, Peter Campbell, Gad Getz, and Jan O. Korbel, *Data Analysis: Create a Cloud Commons*. 523 Nature, 2015, at 149–151. https://doi.org/10.1038/523149a.

3 Madeleine J. Murtagh, Andrew Turner, Joel T. Minion, Michaela Fay, and Paul R. Burton, *International Data Sharing in Practice: New Technologies Meet Old Governance*, 14 Biopres. Biobank, 3, 2016, at 231–240 http://doi.org/10.1089/bio.2016.0002. See also: Katie M. Saulnier et al., *Benefits and Barriers in the Design of Harmonized Access Agreements for International Data Sharing*, 6 Sci. Data, 2019, at 297. https://doi.org/10.1038/s41597-019-0310-4; Bartha M. Knoppers et al. *Population Genomics: The Public Population Project in Genomics (P3G): a Proof of Concept?*, 16 Eur. J. Hum. Genet., 2008, at 664–665. https://doi.org/10.1038/ejhg.2008.55.

4 G.A. Res. 217 (III) A, Universal Declaration of Human Rights (Dec. 10, 1948) at 12.

5 G.A Res. 2200A (XXI), International Covenant on Civil and Political Rights, (Dec. 16, 1966) at 17.

6 Council of Europe, European Convention for the Protection of Human Rights and Fundamental Freedoms, as amended by Protocols Nos. 11 and 14, (Nov. 4, 1980) ETS 5.

7 Council of Europe, Convention for the Protection of Individuals with Regard to the Automatic Processing of Individual Data, (Jan. 28, 1981) ETS 108.

8 Organization for Economic Cooperation and Development, Guidelines on the Protection of Privacy and Transborder Flows of Personal Data (1980). Later updated in the Organization for Economic Cooperation and Development, Privacy Framework (2013). See also: Organisation for Economic Cooperation and Development (OECD), Recommendation of the Council on Health Data Governance. O.E.C.D Legal Instruments (2017).

9 Council of Europe, Protocol amending the Convention for the Protection of Individuals with regard to Automatic Processing of Personal Data, (Oct 10, 2018), CETS 223, Art. 5 (4) (a).

10
*Ibid*.

11 Council of Europe Treaty Office, Chart of Signatures and Ratifications of Treaty 108 (2021).

12
*Ibid*. The concerned countries are: Argentina, Cabo Verde, Mauritius, Mexico, Morocco, Senegal, Tunisia, Uruguay.

13 Organization for Economic Cooperation and Development, Guidelines on the Protection of Privacy and Transborder Flows of Personal Data, *supra* note 8. Organization for Economic Cooperation and Development, Privacy Framework, *supra* note 8.

14 Commission Directive 95/46, 1995, O.J. (281*)* (EC).

15 Franz-Stefan Gady. EU/U.S, *Approaches to Data Privacy and the ‘Brussels Effect’: A Comparative Analysis*. Georgetown Journal of International Affairs. International Engagement on Cyber IV 2014, at 12–23.

16 Paul Craig and Gráinne de Búrca, EU Law: Text, Cases, and Materials. ‘Chapter 8: The Nature and Effect of EU Law: Direct Effect and Beyond’ (7th ed. Oxford University Press, 2020).

17
*Ibid*.

18 Commission Regulation (2016/679), 2016 O.J. (119) (E.C.) at 3 (hereinafter General Data Protection Regulation).

19 European Data Protection Board Guidelines 3/2018, at 5.

20 General Data Protection Regulation, *supra* note 18, at 17.

21 The right to be forgotten was previously enshrined in European data protection law as a result of case law rather than being a formal legislative guarantee. See: Case C-131/12, Google Spain SL and Google Inc. v Agencia Española de Protección de Datos (AEPD) and Mario Costeja González. ECLI:EU:C: 2014:317.

22 European Data Protection Supervisor Opinion 7/2015, Meeting the Challenges of Big Data: A Call for Transparency, User control, Data Protection by Design and Accountability (2015) at 5.

23 General Data Protection Regulation, *supra* note 18, at art. 20.

24
*Ibid* at art. 25.

25 Lee A. Bygrave, *Data Protection by Design and by Default: Deciphering the EU’s Legislative Requirements* 4:2 Oslo L Rev., 2017, at 105.

26 General Data Protection Regulation, *supra* note 18, at art. 35.

27
*Ibid* at art. 30.

28 Graham Greenleaf and Bertil Cottier. *2020 Ends a Decade of 62 New Data Privacy Laws*. 163 Privacy Laws & Business International Report, 2020 at 24.

29 Health Information Portability and Accountability Act, 1996, 45 C.F.R. §§ 160, 164.

30 Financial Services Modernization Act of 1999, (Pub. L. 106–102) 16 C.F.R. § 313.

31 Adrian Thorogood, *Canada: Will Privacy Rules Continue to Favour Open Science?* 137 Hum. Genet., 2018, at 595. https://doi.org/10.1007/s00439-018-1905-0

32 Fruzsina Molnár-Gábor, *Data Protection*, in: Max Planck Encyclopedia for Comparative Constitutional Law (Rainer Grote, Frauke Lachenmann, Rüdiger Wolfrum eds., Oxford University Press, 2017).

33 Hannah Kim, So Yoon Kim, and Yann Joly, *South Korea: in the Midst of a Privacy Reform Centered on Data Sharing*. 137 Hum. Genet., 2018, at 627. https://doi.org/10.1007/s00439-018-1920-1.

34 Michael Kirby, *The History, Achievement, and Future of the 1980 OECD Guidelines on Privacy*, 1 International Data Privacy Law, 2011, at 6. https://doi.org/10.1093/idpl/ipq002.

35 Dara Hallinan, Alexander Bernier, Anne Cambon-Thomsen, et al., *International Transfers of Personal Data for Health Research Following Schrems II: A Problem in Need of a Solution*. Eur. J. Hum. Genet., 2021. https://doi.org/10.1038/s41431-021-00893-y; Marc Rotenberg, *Schrems II, from Snowden to China: Toward a New Alignment on Transatlantic Data Protection*, 26 Eur. Law J., 2020, at 141; Franz-Stefan Gady, *supra* note 15.

36 Matthias Bauer, Martina F. Ferracane et al., Unleashing Internal Data Flows in the EU: An Economic Assessment of Data Localisation Measures in the EU Member States, ECIPE Policy Brief, No. 3/2016, European Centre for International Political Economy (ECIPE), Brussels (2016).

37 General Data Protection Regulation, *supra* note 18, at art. 9.

38 Alexander Bernier, Bartha M. Knoppers, *Health Data Sharing in Canada* 41 Health Law in Canada, 2020, 30 at 44–45.

39 Yongxi Chen and Lingqiao Song, China: *Concurring Regulation of Cross-Border Genomic Data Sharing for Statist Control and Individual Protection*. 137 Hum. Genet., 2018, at 605. https://doi.org/10.1007/s00439-018-1903-2.

40 Alexander Savelyev, *Russia’s New Personal Data Localization Regulations: A Step Forward or a Self-Imposed Sanction?* 32 Comput. L. Secur. Rev., 2016, at 128 https://doi.org/10.1016/j.clsr.2015.12.003.

41
Noela J. Inions, Leanne E. Tran, and Lorne E. Rozovsky, Canadian Health Information: A Practical Legal and Risk Management Guide (4th ed., 2018), LexisNexis Canada.

42 To our knowledge, no exhaustive review of the world’s data localization laws, nor a review of the more circumscribed data localization requirements enshrined in data protection or data privacy statutes, has thus far been performed.

43 Art. 4(3) Digital Health Applications Ordinance of 8 April 2020 (BGBl. I p. 768), as amended by Article 1 of the Ordinance of 22 Sept. 2021 (BGBl. I p. 4355).

44 Cf. information provided by the Federal Institute for Drugs and Medical Devices on the admissibility of data processing outside Germany in connection with the Institute’s review procedure pursuant to Section 139e of the Fifth Book of the German Social Code (SGB V), as of May 31, 2021.

45
*Ibid*.

46 European Medicines Agency. European Medicines Agency Policy on Publication of Clinical Data for Medicinal Products for Human Use (2019); Health Canada, Guidance Document on Public Release of Clinical Information (2019).

47 Bernier and Knoppers, Health Data Sharing in Canada, *supra* note 38.

48 European Commission. Adequacy Decisions, (n.d.), https://ec.europa.eu/info/law/law-topic/data-protection/international-dimension-data-protection/adequacy-decisions_en (accessed Oct. 5, 2021).

49 Commission Implementing Decision (EU) 2019/419 of Jan. 23, 2019 pursuant to Regulation (EU) 2016/679 of the European Parliament and of the Council on the adequate protection of personal data by Japan under the Act on the Protection of Personal Information (Text with EEA relevance), 2019 304 O.J. (L 7), (hereinafter Japan Adequacy Decision).

50 General Data Protection Regulation, *supra* note 18, at art. 49.

51 Case C-362/14, Maximillian Schrems v Data Protection Commissioner ECLI:EU:C:2015:650 (hereinafter Schrems I).

52 Case C-311/18, Data Protection Commissioner v. Facebook Ireland Ltd, Maximillian Schrems ECLI:EU:C:2020:559.

53 Schrems I, *supra* note 51, at paras 90–94.

54
*Ibid.,* at paras 89–95.

55 Case C-311/18, *Data Protection Commissioner v. Facebook Ireland Ltd, Maximillian Schrems,* ECLI:EU:C:2020:559 at a para 138 (hereinafter Schrems II).

56 European Data Protection Board, Recommendations 02/2020 on the European Essential Guarantees for Surveillance Measures (2020).

57
*Ibid.,* at p. 4. Such jurisprudence interprets the Charter of Fundamental Rights of the European Union, especially arts. 7, 8, 47, and 52.

58
*Ibid.,* at p. 4. Such jurisprudence interprets the European Convention on Human Rights, especially art. 8.

59
*Ibid*., at p. 8. The *European Essential Guarantees* are as follows (direct quote): A. *Processing should be based on clear, precise, and accessible rules*; B. *Necessity and proportionality with regard to the legitimate objectives pursued need to be demonstrated*; C. *An independent oversight mechanism should exist*; D. *Effective remedies need to be available to the individual.*

60 Schrems II, *supra* note 55, at paras 134–135.

61 European Data Protection Board, Recommendations 02/2020 on the European Essential Guarantees for Surveillance Measures, *supra* note 56.

62
*Ibid*.

63 Christopher Kuner, Transborder Data Flows and Data Privacy Law at 125–129 (2013).

64 Schrems II, *supra* note 55.

65 General Data Protection Regulation, *supra* note 18, at arts. 40, 46 (2) (e).

66 World Medical Association [WMA], Declaration of Helsinki: Ethical Principles for Medical Research Involving Human Subjects (2013).

67 Council for International Organizations of Medical Sciences [CIOMS] in collaboration with the World Health Organization [WHO], International Ethical Guidelines for Health-Related Research Involving Humans (2016).

68 International Council for Harmonisation of Technical Requirements for Pharmaceuticals for Human Use, Harmonised Clinical Guideline (2016).

69 World Medical Association [WMA], Declaration of Taipei on Ethical Considerations Regarding Health Databases and Biobanks (2016).

70
Tom L. Beauchamp and James F. Childress, Principles of Biomedical Ethics (8th ed. 2019) Oxford University Press.

71 Dana J. Lawrence, *The Four Principles of Biomedical Ethics: A Foundation for Current Bioethical Debate*. 14 J. Chiropr. Humanit., 2007, at 34.

72 Nchangwi Syntia Munung, Bongani M. Mayosi, Jantina de Vries, *Equity in International Health Research Collaborations in Africa: Perceptions and Expectations of African Researchers*. 12 PloS One, 2017, at e0186237. DOI: https://doi.org/10.1371/journal.pone.018623.

73 Arthur W. Toga et al. *Big Biomedical Data as the Key Resource for Discovery Science*, 22 Journal of the American Medical Informatics Association, 2015, at 1126 https://doi.org/10.1093/jamia/ocv077.

74 Ciara Staunton and Jantina de Vries, *The Governance of Genomic Biobanking Research in Africa: Reframing the Regulatory Tilt*, 7 J. L. Biosci., 2020, at 1 DOI: http://dx.doi.org/10.1093/jlb/lsz018. Munung, Mayosi, and de Vries, *supra* note 75, at 59. Susan Bull, *Ensuring Global Equity in Open Research*. Wellcome Trust, 2016 https://dx.doi.org/10.6084/m9.figshare.4055181.

75 Jorge L. Contreras and Bartha M. Knoppers, *The Genomic Commons*, Annu. Rev. 19 Genom. Hum. Genet., 2018, at 429.

76
*Ibid.,* at pp. 438–439.

77 Jorge L. Contreras, *Bermuda’s Legacy: Policy, Patents and the Design of the Genome Commons*, 12 Minn. J. L., Sci. Technol., 2011, at 61.

78 Wellcome Trust. Sharing Data from Large-scale Biological Research Projects: A System of Tripartite Responsibility (2003).

79 Toronto International Data Release Workshop Authors. *Prepublication Data Sharing*. 461 Nature, 2009, at 168. https://doi.org/10.1038/461168a.

80 Contreras, *supra* note 77.

81 United Nations Educational, Scientific, and Cultural Organization [UNESCO]. Recommendation on Open Science (Draft) CL 4363 (2021).

82
*Ibid*.

83 United Nations Educational, Scientific, and Cultural Organization [UNESCO], International Declaration on the Human Genome and Human Rights (1997).

84 United Nations Educational, Scientific, and Cultural Organization [UNESCO], International Declaration on Human Genetic Data (2003).

85 United Nations Educational, Scientific, and Cultural Organization [UNESCO], Universal Declaration on Bioethics and Human Rights (2005).

86 Organisation for Economic Cooperation and Development (OECD), Guidelines for Human Biobanks and Genetic Research Databases (HBGRDs) (2009).

87 Recommendation CM/Rec (2016) 6 of the Committee of Ministers to member States on Research on Biological Materials of Human Origin (CoE).

88 UNESCO, International Declaration on the Human Genome and Human Rights, *supra* note 83, at art. 19. UNESCO, International Declaration on Human Genetic Data, *supra* note 84, at art. 18. Recommendation CM/Rec (2016) 6 of the Committee of Ministers to member States on Research on Biological Materials of Human Origin, *supra* note 87 (Preamble).

89 UNESCO, International Declaration on Human Genetic Data, *supra* note 84, at art. 19.

90
*Ibid*. See also: Rumiana Yotova, Bartha M Knoppers, *The Right to Benefit from Science and Its Implications for Genomic Data Sharing*, 31 Eur. J. Int. L., 2020, at 665. https://doi.org/10.1093/ejil/chaa028.

91 UNESCO, International Declaration on the Human Genome and Human Rights, *supra* note 83, at art. 5 (b), 5 (e). See also: UNESCO, International Declaration on Human Genetic Data, *supra* note 84, at art. 6.

92 UNESCO, International Declaration on the Human Genome and Human Rights, *supra* note 83, at art. 53 (c). See also: UNESCO, International Declaration on Human Genetic Data, *supra* note 84, at art. 10.

93 UNESCO, International Declaration on the Human Genome and Human Rights, *supra* note 83, at arts. 5 (d), 7. See also: UNESCO, International Declaration on Human Genetic Data, *supra* note 84, at art. 6.

94 UNESCO, International Declaration on the Human Genome and Human Rights, *supra* note 83, at arts. 5 (d) (7). See also: UNESCO, International Declaration on Human Genetic Data, *supra* note, 84 at art. 6.

95 UNESCO, International Declaration on Human Genetic Data, *supra* note 84, at art. 13–14.

96
*Ibid* at arts. 8, 14.

97
*Ibid* at art. 2, art. 14 (c).

98
*Ibid* at art. 9 (b).

99
*Ibid* at art. 16 (b).

100 International Electrotechnical Commission. What is an International Standard? (n.d.), https://www.iec.ch/understanding-standards (accessed Oct. 5, 2021).

101 Bartha M. Knoppers, *Framework for Responsible Sharing of Genomic and Health-Related Data*, 8, Hugo J., 2014, at 3.

102 Global Alliance for Genomics and Health, Framework for Responsible Sharing of Genomic and Health-Related Data (2014).

103 Global Alliance for Genomics and Health, Consent Policy (2019).

104 Global Alliance for Genomics and Health, Copyright Policy (2020).

105 Global Alliance for Genomics and Health, Data Privacy and Security Policy (2019).

106 Global Alliance for Genomics and Health, Accountability Policy (2016).

107 Global Alliance for Genomics and Health, Ethics Review and Recognition Policy (2020).

108 Global Alliance for Genomics and Health, Genomic Data Toolkit (n.d.).

109 Global Alliance for Genomics and Health, Regulatory and Ethics Toolkit (n.d.).

110 Global Alliance for Genomics and Health, Data Security Toolkit (n.d.).

111 Harnessing Genomic Technologies Toward Improving Health in Africa: Opportunities and Challenges. Recommendations for the Health in Africa (H3 Africa) Initiative to the Wellcome Trust and the National Institutes of Health (2011).

112 H3 Africa, Ethics and Governance Framework for Best Practice in Genomic Research and Biobanking in Africa (2017).

113 H3 Africa, Guideline for Informed Consent, Third Edition (2018).

114 H3 Africa, Publications Policy: Human Heredity and Health in Africa (2014).

115 H3 Africa, Guideline for the Return of Individual Genetic Research Findings (2018).

116
*Ibid*.

117 Andrew Treolar, *The Research Data Alliance: Globally Co-ordinated Action Against Barriers to Data Publishing and Sharing* 27 Learn. Publ. S9, 2014, at S10 DOI: http://dx.doi.org/10.1087/20140503.

118 Mark A. Parsons, *The Research Data Alliance: Implementing the Technology, Practice, and Connections of a Data Infrastructure* 39 Bull. Am. Soc. Inf. Sci. Technol. 33, 2013, at 35. DOI: http://dx.doi.org/10.1002/bult.2013.1720390611.

119 Research Data Alliance. Outputs and Recommendations (n.d.), https://rd-alliance.org/recommendations-and-outputs/catalogue (accessed Oct. 5, 2021).

120 Example of an international treaty: Council of Europe, Protocol amending the Convention for the Protection of Individuals with regard to Automatic Processing of Personal Data, (10 Oct) 2018, CETS 223. Example of a non-binding legal instrument: Organisation for Economic Cooperation and Development (OECD), Recommendation of the Council on Health Data Governance. O.E.C.D Legal Instruments (2017).

121 General Data Protection Regulation, *supra* note 18, at arts. 40, 42, and 47. Phillips, M., Molnár-Gábor, F., Korbel, J.O., Thorogood, A., Joly, Y., Chalmers, D., Townend, D., & Knoppers, B.M., *Genomics: Data Sharing Needs an International Code of Conduct*, 578 Nature, 2020, at: 31–33.

122 The Human Cell Atlas Ethics Toolkit, https://www.humancellatlas.org/ethics/. The International Cancer Genome Consortium Accelerating Research in Genomic Oncology (ICGC-ARGO) Policies and Guidelines, https://www.icgc-argo.org/page/75/e1-ethics-and-informed-consent (accessed Oct. 5, 2021).

123 Saulnier et al., *supra* note 3. Alexander Bernier, Adrian Thorogood, Sharing Bioinformatic Data for Machine Learning, Maximizing Interoperability through License Selection Proceedings of BIOSTEC 2020: 13th International Joint Conference on Biomedical Engineering Systems and Technologies, Bioinformatics 2020: International Conference on Bioinformatics Models, Methods and Algorithms (2020). DOI: http://dx.doi.org/10.5220/0009179502260232.

124 European Data Protection Board, Guidelines 1/2019 on Codes of Conduct and Monitoring Bodies under Regulation 2016/679 (2019). European Data Protection Board, Guidelines 04/2021 on Codes of Conduct as Tools for International Transfers (2021). Reliance on co-regulation models (i.e., mixed public and private engagement in devising and enforcing rules) is compatible with theoretical literature on the microbial commons, which recognizes that neither central government acts, nor pure private self-governance efforts, can lead to the efficient development of stable governance arrangements for international microbial commons. See: Jerome H. Reichman, Paul F. Uhlir, and Tom Dedeurwaerdere, Governing Digitally Integrated Genetic Resources, Data, and Literature (2017, Cambridge University Press).

125 General Data Protection Regulation, *supra* note 18 at arts. 32, 46.

126 Alexander Bernier, Rare Disease Data Stewardship in Canada. 5 FACETS 836 https://doi.org/10.1139/facets-2020-0050; Global Alliance for Genomics and Health: Data Use Ontology. (n.d.), https://www.ga4gh.org/news/data-use-ontology-approved-as-a-ga4gh-technical-standard/ J. Patrick Woolley, Emily Kirby, Josh Leslie et al., *Responsible Sharing of Biomedical Data and Biospecimens via the Automatable Discovery and Access Matrix(ADA-M)*, 3 npj Genomic Med., 2018, at 17. https://doi.org/10.1038/s41525-018-0057-4; Stephanie O.M. Dyke et al., *Consent Codes: Upholding Standard Data Use Conditions*. 12 PLoS Genet., 2016, at e1005772 https://doi.org/10.1371/journal.pgen.1005772.

127 Lisa M. Austin and David Lie, *Safe Sharing Sites*. 94 N.Y.U. L Rev., 2019, at 4.

128 Marilyn J. Borugian, *The Canadian Partnership for Tomorrow Project: Building a Pan-Canadian Research Platform for Disease Prevention*. 182 CMAJ, 2010, at 1197, https://doi.org/10.1503/cmaj.091540.

129 Bartha Maria Knoppers and Yann Joly, *Introduction: the Why and Whither of Genomic Data Sharing*, 137 Hum. Genet., 2018, at 569. https://doi.org/10.1007/s00439-018-1923-y.

130 Autism Speaks, MSSNG Database for Autism Researchers.

131 Adrian Thorogood, *Policy-Aware Data Lakes: A Flexible Approach to Achieve Legal Interoperability for Global Research Collaborations*, 7 J. L. Biosci., 2020, https://doi.org/10.1093/jlb/lsaa065.

132 Susan E. Wallace, Emily Kirby and Bartha M. Knoppers. *How Can We Not Waste Legacy Genomic Research Data?* 11 Front. Genet., 2020, at 446, https://doi.org/10.3389/fgene.2020.00446

133
*Ibid*.

134 Marc Fiume et al., *Federated Discovery and Sharing of Genomic Data Using Beacons*, 37 Nat. Biotechnol., 2019, at 220, https://doi.org/10.1038/s41587-019-0046-x.

135 Froelicher, D., Troncoso-Pastoriza, J. R., Raisaro, J. L., Cuendet, M. A., Sousa, J. S., Cho, H., Berger, B., Fellay, J., & Hubaux, J. P., *Truly Privacy-Preserving Federated Analytics for Precision Medicine with Multiparty Homomorphic Encryption*, 12 Nat. Commun. 2021, at 5910. https://doi.org/10.1038/s41467-021-25972-y.

136 Adrian Thorogood et al., *International Federation of Genomic Medicine Database Using GA4GH Standards* 1 Cell Geonomics, 2021. See also: Christine Suver, Adrian Thorogood, Megan Doerr, John Wilbanks, and Bartha Knoppers, *Bringing Code to Data: Do Not Forget Governance*, 22 J. Med. Internet Res., 2020, at e18087.

137 Government of Canada, Privacy Enhancing Technologies—A Review of Tools and Techniques: Report prepared by the Technology Analysis Division of the Office of the Privacy Commissioner of Canada (2017).

138 Alexander Bernier, *supra* note 126.

139
*Ibid*.

140 The ICGC Data Access Compliance Office and the ICGC International Data Access Committee, et al., *Analysis of Five Years of Controlled Access and Data Sharing Compliance at the International Cancer Genome Consortium*, 48 Nat. Genet., 2016, at 224, https://doi.org/10.1038/ng.3499

141
*Ibid*.

142 Saulnier, *supra* note 3.

143
*Ibid*.

144
*Ibid*.

145 Ira S. Rubinstein, *Regulating Privacy by Design*, 26 Berkeley Technol. L. J., 2012, at p. 1409, 1420–1423. Ira S. Rubinstein and Nathaniel Good, *Privacy by Design: A Counterfactual Analysis of Google and Facebook Privacy Incidents* 28 Berkeley Technol. L. J., 2013, at 1333.

146 Rubinstein, *supra* note 145, at 1420–1423.

147
*Ibid*.

148
*Ibid*.

149 General Data Protection Regulation, *supra* note 18, at art. 25.

150
*Ibid* at art. 32.

151 Cynthia Dwork and Aaron Roth, *The Algorithmic Foundations of Differential Privacy*, 9 Found. Trends Theor. Comput. Sci., 2014, at 211.

152 Joseph Ficek, Wei Wang, Henian Chen, Getachew Dagne, Ellen Daley, *Differential Privacy in Health Research: A Scoping Review*, 28 J. Am. Med. Inform. Assoc., Oct. 2021, at 2269–2276, https://doi.org/10.1093/jamia/ocab135. Simmons S. and Berger B., *Realizing Privacy Preserving Genome-Wide Association Studies* 32 Bioinformatics, 2015, at 1293. Simmons S, Sahinalp C., and Berger B., *Enabling Privacy-Preserving GWASs in Heterogeneous Human Populations*, 3 Cell Systems, 2016, at 54. Dankar, F. K., & El Emam, K. *Practicing Differential Privacy in Health Care: A Review*. 6 Trans. Data Priv., 2013, at 35–67.

153 Global Alliance for Genomics and Health, Beacon Project (Beacon API v. 2.0), https://beacon-project.io/. See also: Diyue Bu, *Human Genomic Sharing and Privacy*. Doctoral Thesis. Indiana University, Luddy School of Informatics ProQuest Dissertations Publishing, 2021. Zhiyu Wan et al. *Controlling the Signal: Practical Privacy Protection of Genomic Data Sharing through Beacon Services*. 10 BMC Med. Genomics, 2017, at 39. https://doi.org/10.1186/s12920-017-0282-1 (accessed Oct. 5, 2021).

154 Government of Canada, *supra* note 137.

## Appendix 1. Policy Documents of the Global Alliance for Genomics and Health (GA4GH), the Research Data Alliance (RDA), and Human Health and Heredity in Africa (H3 Africa)

**Table A1:** Organization descriptions and associated documents and policies

Organization	Policy documents
Global Alliance for Genomics and Health	**Nature of organization:** The GA4GH is not affiliated with a singular research consortium or institution, but instead is composed of members from multiple research institutions that attempt to foster harmonization and the development of common standards throughout the wider research community. The GA4GH describes itself as follows: ‘The Global Alliance for Genomics and Health (GA4GH) is a policy-framing and technical standards-setting organization, seeking to enable responsible genomic data sharing within a human rights framework’. **Categories of documents:** Toolkits, policies, and standards that health consortia or health research institutions can voluntarily adopt as best practices for ensuring health data sharing, regulatory and ethics compliance, and data security. **Documents:** The Framework for Responsible Sharing of Genomic and Health-Related Data (2014) **Toolkits:** Genomic Data Toolkit Regulatory and Ethics Toolkit Data Security Toolkit **Select policies and standards:** Accountability Policy Consent Clauses for Genomic Research Consent Policy Copyright Policy Data Access Committee Review Standards (DACReS) Policy Data Privacy and Security Policy Ethics Review Recognition Policy Machine Readable Consent Guidance Model Consent Clauses for Rare Disease Research Pediatric Consent Clauses **Select technical tools:** Beacon API Crypt4GH Data Use Ontology (DUO) GA4GH Passports
Research Data Alliance	**Nature of organization:** The Research Data Alliance (RDA) is a The RDA solicits the creation of decentralized volunteer working groups of temporary duration composed of community members and experts from multiple institutions and scientific domains. These groups ideate and develop deliverables and outputs, which are subject to approval and publication by central RDA leadership. The RDA also supports the creation of groups of indefinite duration that are less structured in their composition, and are not required to produce documents. **Categories of documents:** The Research Data Alliance recognizes three general categories of work products, which Working Groups create and publish. The first, Recommendations, are published conditional on approval from central RDA leadership. The second are Supporting Outputs and Other Outputs, which usually serve as documentation that is associated with a corresponding RDA Recommendation. These include surveys, research publications, and summary guidance documents. Last, Standards are formal standards that are submitted from the RDA to the European Multi-Stakeholders Platform on ICT Standardization (MSP—E02758) for formal approval as European Technical Standards. The Research Data Alliance has coordinated with this European standards-setting organization (EMSP) to submit select work-products for formal endorsement through its standards-setting procedures. **Select recommendations:** FAIR Principles for Research Software (FAIR4RS Principles) Machine Actionable Policy Templates – Practical Policy WG Recommendations Guidelines for Publishing Structured Metadata on the Web RDA COVID-19 Recommendations and Guidelines for Data Sharing
Human Heredity and Health in Africa (H3 Africa)	**Nature of organization:** Human Heredity and Health in Africa (H3 Africa) is an international research consortium that is principally engaged in research infrastructure development and fundamental research on diseases in the African continent. The consortium comprises 51 research projects, which African investigators coordinate and lead. Developing generalizable ethics, legal, and policy guidance for researchers in Africa is an objective of the consortium. **Categories of documents:** The H3 Africa consortium has produced an Ethics and Governance Framework that is intended for biobanking initiatives and genomic research performed in Africa. The consortium has also developed a number of consortium-specific guidelines, template documents, and policies. **Select governance documents and policies:** Ethics and Governance Framework for Best Practice in Genomic Research and Biobanking in Africa H3 Africa Consortium: Guideline for Informed Consent Publications Policy: Human Heredity and Health in Africa (H3 Africa) H3 Africa: Guidelines for Community Engagement H3 Africa: Data and Biospecimen Access Committee Guideline

## Appendix 2. Examples of Consortium Consent Documents and Policies

**Table A2:** Documents and policies by consortium or organization

Consortium or organization	Documents and publications
The Human Cell Atlas	General ethics tools landing page Human Cell Atlas (HCA) Core Consent Elements Human Cell Atlas (HCA) Retrospective Assessment Filter Retrospective Assessment Filter – associated publication Template consent form – main – healthy adult participant Consent form addendum – clinical tissue Template consent form – deceased participant (anatomical gift) Template consent form – deceased participant (ante-mortem consent or post-mortem next-of-kin consent) Template consent form – informed consent to donation of leftover embryos Template consent form – use of fetal tissue for research
The International Cancer Genome Consortium (ICGC) Accelerating Research in Genomic Oncology (ARGO).	ICGC ARGO. Core Consent Elements Retrospective Assessment Filter – associated publication. ICGC ARGO. Informed Consent, Access, and Ethical Oversight ICGC ARGO. Ethics and Informed Consent
Canada’s National COVID-19 Immunity Task Force (CITF)	COVID-19 Immunity Task Force, Prospective Consent Guidance (including template informed consent clauses) COVID-19 Immunity Task Force, Retrospective Consent Guidance (including retrospective consent filter)
Canada Personal Genome Project (PGP).	General consent materials landing page Personal Genome Project Canada - Eligibility Screening Consent Form Personal Genome Project Canada – Main Consent Form
Harvard Personal Genome Project (PGP).	General consent materials landing page Harvard Personal Genome Project – Eligibility Screening Consent Form Harvard Personal Genome Project – Main Consent Form

## Appendix 3. Examples of Data Access Controls

**Table A3:** Data access: tiers, bodies, policies by consortium or database

Consortium or database	Data access tiers	Data access bodies and governance structures.	Access policies and governance documentation
Canadian Longitudinal Study on Aging (CLSA)	Controlled access database.	CLSA Data and Sample Access Committee (DSAC) CLSA Governance structure	Flowchart of CLSA data access processes Data and Biospecimen Access Policy and Guiding Principles Data Access Agreement.
Canadian Partnership for Tomorrow’s Health (CANPath).	Controlled access database. Access requests for data from singular participating regional cohorts are coordinated through the access procedures of the concerned local cohorts. Access requests for data from multiple participating regional cohorts are coordinated through the central CANPath Data Access Committee (DAC).	CANPath Governance structure	Regional CanPath Cohorts Canadian Partnership for Tomorrow’s Health (CANPath) Access Policy CanPATH Guidelines for Biosample Access
NHGRI Analysis Visualization and Informatics Lab-Space (AnVIL).	Open access / registered access (i) and controlled access (ii) database. The most permissive tier of data access is described as ‘open access,’ albeit it requires user registration on the platform website.	NHGRI AnVIL Leadership Team Each cohort participating in NHGRI AnVIL manages access to its data independently, though select consortia have entered into contractual agreements to recognise their respective data access approvals on a consortium-wide basis (i.e., approval by one consortium partner’s DAC qualifies the applicant for access to the data belonging to all consortium partners).	NHGRI AnVIL: Requesting Data Access
Database of Genotypes and Phenotypes (dbGaP).	Controlled access database. A traditional controlled access process is applicable to most dbGaP data. A simplified controlled access process is applied to select data that is authorized for General Research Use (GRU).	Each cohort participating in dbGaP manages access to its data independently, through its own data access committee (DAC). The dbGaP controlled access process utilises two paths to data access, one for NIH staff member applicants and another for NIH-external applicants.	dbGaP Overview dbGaP Data Download dbGaP Tips for Preparing a Successful Data Access Request NIH Extramural Data Access Request Flowchart NIH Staff Data Access Request Flowchart NIH Staff Request form for Permission to Access Controlled-Access Data
International Cancer Genome Consortium (ICGC).	Controlled access database. The ICGC Data Access Compliance Office (DACO) and Data Access Committee (DAC) administer requests for most ICGC controlled-access data. Access to U.S.-source ICGC datasets is instead coordinated through dbGaP.	ICGC ARGO – Structure and Governance ICGC Data Access Control Bodies ICGC Data Access Compliance Office (DACO)	ICGC ARGO – Applying for Access to Controlled Access Data ICGC ARGO – Data Access and Data Use Policies and Guidelines ICGC ARGO – Data Access Framework ICGC ARGO – International Data Sharing ICGC ARGO – Ethics and Informed Consent
U.K. Biobank.	Controlled access database.	U.K. Biobank. Governance U.K. Biobank Access Sub-Committee	U.K. Biobank Access Procedures: Access and Review Procedures for Access to the U.K. Biobank Resource U.K. Biobank. Data Management and Sharing Plan U.K. Biobank Re-contact Procedures for Third-Party Researchers U.K. Biobank. Frequently asked questions on applying to and accessing U.K. Biobank Data U.K. Biobank Governance Framework U.K. Biobank Original Research Protocol (2007)
PhenomeCentral.	Registered access database. PhenomeCentral is a registered-access platform that enables clinicians to ‘match’ information concerning local cases of unnamed rare diseases to other cases presenting similar genotypes and/or phenotypes that are identified at other clinical centres.		PhenomeCentral Data Policies PhenomeCentral Consent Forms
Database of Chromosomal Imbalance and Phenotype in Humans Using Ensembl Resources (DECEIPHER)	Registered access to patient reports (access is restricted to consortium participants). Open access to anonymised positional genomic information and information concerning the related phenotype.	Publication describing the access model of the DECEIPHER platform	DECEIPHER platform public-facing description, detailing distinct tiers of data access.
The Human Cell Atlas (HCA)	Open access and controlled access database. The Human Cell Atlas is a database containing allele count matrices, select single-cell RNAseq data, and associated metadata. The Human Cell Atlas contains a public open-access tier of data access, and is in the process of implementing a controlled access tier of data access for restricted or highly sensitive datasets.	Human Cell Atlas Governance Human Cell Atlas Working Groups	Human Cell Atlas Open-Access Data Use Agreement Human Cell Atlas Ethics Toolkit
Personal Genome Project.	Open access database.		Personal Genome Project Guidelines

## Appendix 4. Consortium Working Groups and Expert Bodies

**Table A4:** Working groups and expert bodies by consortium

**Consortium**	**Examples of working groups or other expert bodies**	**Purpose**
dbGaP	dbGaP Help Desk	The dbGaP help desk is a queries portal through which researchers, including external researchers applying for access to controlled-access dbGaP data, can request assistance from, or submit queries to, dbGaP staff members.
Human Cell Atlas	Organizing Committee	The Organizing Committee is the central HCA body responsible for executive decision-making. This body is comprised of esteemed scientific experts from research institutions world-wide.
Human Cell Atlas	The Human Cell Atlas Equity Working Group (EqWG)	The Equity Working Group champions equity in the work of the Human Cell Atlas. The work of this group includes proposing, supporting, and developing initiatives to enable the participation of researchers that are members of geographic, ethnic, and other groups which have traditionally been excluded from equitable participation in, and benefit from, biomedical research.
Human Cell Atlas	The Human Cell Atlas thics Working Group (EWG) The Human Cell Atlas Ethics Helpdesk	The Ethics Working Group is comprised of experts in the law, international bioethics norms, and related disciplines in the social sciences. This working group provides interdisciplinary input on the development of bioethics deliverables such as contracts, forms, policies, platforms and other related organisational and technological tools. The HCA Ethics Helpdesk is a point of contact for researchers intending to deposit data to the HCA or to access and use HCA data. The help desk provides general information regarding international and local bioethics norms, and regarding relevant HCA ethics policies.
Human Cell Atlas	The Human Cell Atlas Analysis Working Group	The Analysis Working Group is responsible for supporting the development of policies regarding analysis methodologies and recommended practices for technical aspects of HCA data analysis.
CanCOGeN HostSeq and CanCOGeN VirusSeq.	CanCOGeN Steering Committee	The Canadian COVID-19 Genomics Network (CanCOGeN) project is divided into two principal branches, HostSeq and VirusSeq, which are concerned with data collection and data dissemination of host genomic data and virus genomic data, respectively. The Steering Committee is responsible for the oversight and coordination of the overall initiative, as well as ensuring its interoperability with similar external projects.
CanCOGeN HostSeq and CanCOGeN VirusSeq.	CanCOGeN Coordinating Committee	The Coordinating Committee works to coordinate efforts and practices regarding elements such as data management and metadata management, across both branches of CanCOGeN, and with select external efforts.
CanCOGeN VirusSeq.	CanCOGeN VirusSeq Implementation Committee	The Implementation Committee ensures the alignment of VirusSeq implementation with societal imperatives including experience, bioethics norms, and the furtherance of equitable outcomes.
CanCOGeN HostSeq.	CanCOGeN HostSeq Implementation Committee	The Implementation Committee ensures the alignment of HostSeq implementation with societal imperatives including experience, bioethics norms, and the furtherance of equitable outcomes.
CanCOGeN HostSeq.	Data Storage and Sharing Sub-Committee	This sub-committee is responsible for enabling the storage and accessibility of CanCOGeN HostSeq data, and for enabling the infrastructure and policy developments required to enable data discoverability and linkage of relevant datasets.
CanCOGeN VirusSeq.	Ethics and Governance Working Group	The Ethics and Governance Working Group participates in the development of ethical, legal, and governance activities to enable the sharing of VirusSeq data with the general the public, with public health authorities, and with researchers in the health sector.
CanCOGeN VirusSeq.	Metadata Working Group	The metadata Working Group is responsible for ensuring that CanCOGeN data and associated metadata is formatted and structured such as to allow it to be meaningfully compared to other related datasets in the Canadian research ecosystem and outside thereof.
CanCOGeN VirusSeq.	Research Collaborations Working Group	The research collaborations working group is responsible for facilitating research collaborations between CanCOGeN VirusSeq and external research groups, and for ensuring the representation of a wide range of Canadian research projects and international research projects in CanCOGeN VirusSeq.
U.K. Biobank.	U.K. Biobank Board of Directors	The Board of Directors is responsible for the executive functioning and operations of the U.K. Biobank.
U.K. Biobank.	International Scientific Advisory Board	The International Scientific Advisory Board is responsible for ensuring the alignment of the U.K. Biobank’s activities with the scientific demands of the international research community and the furtherance of public health objectives.
U.K. Biobank.	Ethics Advisory Committee	The Ethics Advisory Committee is responsible for refining the ethics and ethics-related policies of the U.K. Biobank through time, for engaging in discussion of relevant bioethics-related issues that arise, and for conducting and publishing research relating to salient issues in research ethics and bioethics.
U.K. Biobank.	Access Sub Committee	The Access Sub Committee of the U.K. Biobank Board of Directors is formally responsible for allocating access to U.K. Biobank data and biomaterials. The U.K. Biobank Principal Investigator or their designate is responsible for making determinations regarding the allocation of access to U.K. Biobank data and biomaterials. Specific queries or concerns in administering access to the resource are referred from the U.K. Biobank Principal Investigator to the Access Sub Committee.
U.K. Biobank.	Expert Working Groups	The U.K. Biobank has implemented a Genetics Expert Working Group, and an Imaging Working Group. The Expert Working Groups assist the U.K. Biobank Executive Team in developing and implementing methodologies for the collection of data, especially large datasets.

## Appendix 5. Categories of Consortium Agreements and Consortium Policies

**Table A5:** Explanatory overview of categories of contracts and agreements

Categories of agreements and policies	Purpose
Template Material and Data Transfer Agreement (MDTA)	A research consortium often releases a template Material and Data Transfer Agreement (MDTA) to enable research collaborators that intend to contribute data to the database to share biomaterials or data amongst themselves for sequencing and/or analysis purposes. This template agreement enables research collaborators to establish the conditions of data sharing amongst themselves prior to contribution of the data to the central consortium, whilst still ensuring that the requisite rights and permissions remain in place to enable the contribution thereof to the central consortium.
Data Contribution Agreement/Data Submission Agreement	A Data Contribution Agreement or Data Submission Agreement is a standard-form agreement that a centralized research consortium requires its contributors to sign prior to the upload of data. The purpose of this agreement is to obtain confirmation that the contributing researchers and/or their research institutions hold the necessary ethical and legal rights in the concerned data to submit it to the consortium. The agreement also addresses numerous other matters. These matters sometimes include the respective apportionment of intellectual property rights between the contributor, the consortium, and downstream users in primary and derived discoveries. Also addressed are issues such as the anticipated research attribution that will be afforded to data contributors and to the consortium when the data are used, and the respective guarantees made by the consortium and the contributor (or lack thereof) regarding issues such as data quality, legal rights in data, and the privacy of research participants.
Data Access Agreement	The Data Access Agreement is the corollary of the Data Contribution Agreement or Data Submission Agreement. External researchers must sign such an agreement prior to being granted access to data from a centrally stewarded consortium. Such an agreement generally requires the applicant researchers to attest to having obtained the requisite ethical and legal approvals required for their intended data use, and further attest to their present and ongoing compliance with consortium policies. These policies can include requirements such as the use of appropriate data security measures, preclusions on the re-identification of the individuals that the data concerned, and requirements to avoid using intellectual property rights to stifle further access to the research database. Common terms also include specifications regarding the duration of the applicants’ data access rights, a description of authorized users, and the boundaries and limitations of the applicants’ data usage rights.
Open Access Browse-Wrap Terms-of-Use	For open access datasets, no data access agreement is generally applicable. Some research consortia nonetheless use a browse-wrap terms-of-use to govern access to their open access data. A browse-wrap terms-of-use is not drawn to the attention of the end-user and does not require them to sign or otherwise agree to such contract. In numerous jurisdictions, browse-wrap agreements are not generally found to be legally binding in nature, though this can often be a fact-specific determination.
Open Access Click-Wrap Terms-of-Use	For open access datasets, no data access agreement is generally applicable. Some research consortia nonetheless use a click-wrap terms-of-use to govern access to their open access data. A click-wrap terms-of-use is often drawn to the attention of the end-user and requires them to accept its terms by clicking an acceptance box or otherwise acceding to the agreement. The capacity of a click-wrap agreement to legally bind the end-user is a fact-specific determination and can further be variable from one jurisdiction to another.
Data Attribution Policies	Data attribution policies establish how data users are required or expected to cite or to otherwise provide attribution to the source of such data. Such policies can require or request the attribution of the original research group that generated or contributed the data, the attribution of the consortium or other data repository that hosted the data, or of a specific dataset identifier such as a publication or a digital object identifier (DOI).
Licensing Policies or Intellectual Property Policies	Licensing policies are often used to establish rules or recommendations directed to downstream users of a consortium’s data. These policies sometimes impel downstream data users to adopt I.P. practices concerning the data used that do not interfere with other consortium users’ rights to benefit from the use of such consortium data. More demanding policies might require downstream users to adopt open licensing practices concerning downstream discoveries or derivative datasets, or might require downstream users to contribute such research outputs back to the central consortium database. Some might require data users to explicitly grant I.P. rights in downstream discoveries or in derived datasets back to the central consortium; these should be cautioned against due to their chilling effect on innovators hoping to commercialize their discoveries.

